# Feasibility of Technology-Assisted Lifestyle Self-Monitoring in Older Adults With Type 2 Diabetes: Mixed Methods Pilot Study

**DOI:** 10.2196/79591

**Published:** 2026-06-03

**Authors:** Yan Du, Jing Wang, Byeong Yeob Choi, Helen P Hazuda, Lixin Song

**Affiliations:** 1 School of Nursing, The University of Texas Health Science Center at San Antonio, 7703 Floyd Dr, San Antonio, TX, 78229, United States, 1 210-567-2107; 2 School of Nursing, Florida State University, Tallahassee, FL, United States; 3 Department of Population Health, School of Medicine, The University of Texas Health Science Center at San Antonio, San Antonio, TX, United States; 4 Department of Medicine, School of Medicine, The University of Texas Health Science Center at San Antonio, San Antonio, TX, United States

**Keywords:** digital health, older adults, type 2 diabetes, diabetes self-management, lifestyle intervention

## Abstract

**Background:**

Type 2 diabetes is a major public health concern in older adults. Healthy lifestyles, such as physical activity and healthy eating, are effective strategies for diabetes self-management. Increasing evidence shows that health technologies can promote healthy lifestyles for diabetes management. However, limited research has evaluated their use among older adults with type 2 diabetes.

**Objective:**

This study evaluated the feasibility and preliminary health outcomes of technology-assisted lifestyle monitoring for diabetes management in older adults with type 2 diabetes. The study also examined participants’ experiences to identify barriers and facilitators to sustained technology-assisted lifestyle modification.

**Methods:**

This 12-week pilot study used a pretest and posttest design. Feasibility was assessed by recruitment, retention, and adherence to device-based self-monitoring, including the percentage of days with tracked steps (PDWTs) and the percentage of days with food logs (PDWFLs). Fitbit fitness trackers paired with smartphone apps were used to track physical activity and food intake in 15 overweight/obese older adults with type 2 diabetes (mean age 70.5, SD 4.8 y). Self-monitoring behaviors were tracked throughout the study. Baseline and 12-week health outcomes (eg, hemoglobin A_1c_ [HbA_1c_] and physical function) were compared using paired 2-tailed *t* tests or Wilcoxon signed rank tests, as appropriate; effect sizes were calculated using Cohen *d*. Associations between self-monitoring data (PDWTs, average daily steps, PDWFL) and health outcomes were examined using Pearson or Spearman correlations. Semistructured interviews were conducted at the study completion, and thematic analysis was used to analyze qualitative data.

**Results:**

The target sample (n=15) was successfully enrolled over 6 months, with 100% retention at 12 weeks. Feasibility was supported by consistent use of wearable devices for self-monitoring of physical activity, although dietary logging adherence varied. HbA_1c_ decreased from baseline to 12 weeks (effect size −0.49, 95% CI –1.15 to –0.04; *P*=.04), and PDWFL was inversely correlated with HbA_1c_ (*r*=−0.53; *P*=.04) at follow-up. The qualitative findings indicated that barriers and facilitators to technology-assisted lifestyle self-monitoring and diabetes management through lifestyle modifications exist across multiple levels, including the individual, interpersonal, organizational or community, and societal levels in older adults with type 2 diabetes. Factors at one level interacted with those at other levels. For example, limited technological proficiency challenged lifestyle tracking, while interpersonal and organizational support helped mitigate barriers.

**Conclusions:**

Technology-assisted self-monitoring of lifestyle behaviors was feasible in this sample of older adults with type 2 diabetes and was associated with favorable signals in glycemic control. While causal inferences cannot be drawn from this single-arm pilot study, observed within-subject changes and behavioral-clinical correlations support further evaluation of technology-assisted lifestyle self-monitoring. The findings also highlight the importance of addressing interconnected factors at multiple levels, tailored to older adults’ unique needs and capacities in diabetes self-management.

## Introduction

Diabetes, with over 90% being type 2 diabetes, is a major public health concern. Globally, an estimated 589 million or 1 in 9 adults were living with diabetes in 2025, and the burden is disproportionately affecting older adults [1,2]. In the United States, the prevalence of diabetes is 29.2% in older adults aged 65 years or older based on national data from 2017 to 2020 [3]. Diabetes is strongly associated with adverse health outcomes, including physical disability, cardiovascular disease, physical and cognitive decline, frailty, and chronic kidney disease, leading to substantial individual and societal burden, premature death, and reduced quality of life [4-10]. As the population continues to age rapidly, there is an urgent need to improve type 2 diabetes management and mitigate its associated health burden among older adults.

Lifestyle modification is widely recommended as the first-line management strategy for type 2 diabetes across the lifespan, including in older adults [11,12]. Substantial evidence indicates that regular physical activity combined with healthy eating can improve glycemic control, reduce cardiometabolic risk factors, support modest weight loss, and enhance physical function and quality of life in adults with type 2 diabetes [13-15]. Clinical guidelines further emphasize the importance of individualized lifestyle plans that account for older adults’ functional capacity, comorbidities, and risk of hypoglycemia to ensure safety and enhance adherence [10]. Despite strong recommendations and the known benefits of lifestyle interventions, many older adults face barriers to engagement, including limited mobility, comorbid conditions, and social or environmental constraints, underscoring the ongoing need for tailored, supportive strategies in real-world settings [13,16].

In the digital era, increasing evidence shows that wearable and mobile health devices, such as Fitbit fitness trackers, have the potential to promote healthy lifestyles, such as increased physical activity and weight loss in adults [17,18]. However, reviews highlight that the majority of digital lifestyle intervention studies have focused on younger or middle-aged populations, with limited inclusion of adults aged 65 years and older [14]. Although wearable devices may facilitate lifestyle behavior change by motivating and supporting users [17,19], current evidence suggests that their direct impact on chronic health outcomes is small or nonsignificant [17,20,21]. Beyond technological features, prior studies demonstrate that digital health adoption among older adults is strongly influenced by digital literacy, opportunity, motivation, and the availability of social or structural support [16,22]. These findings underscore the importance of examining not only technological feasibility but also user experience, contextual barriers, and supportive strategies, particularly in light of persistent challenges related to digital literacy, usability, and access among older adults with type 2 diabetes [23-26].

To address these critical gaps in evidence and to inform the development of scalable, age-appropriate digital lifestyle interventions, we conducted a 1-arm, 12-week pilot study among older adults with type 2 diabetes. The objectives of this pretest and posttest study were to (1) assess the feasibility of using health technologies to support self-monitoring of lifestyle behaviors for diabetes management; (2) examine the preliminary changes in health outcomes following technology-assisted lifestyle self-monitoring in older adults with type 2 diabetes; and (3) explore participants’ experiences of using health technologies and identify barriers, facilitators, and potential strategies to support sustained lifestyle modification.

## Methods

### Study Design and Population

This 12-week pilot study used a single-arm, pretest and posttest, mixed methods design. We collected both quantitative and qualitative data, with the quantitative data analyzed to assess study feasibility, self-monitoring behaviors, and within-subject changes in health outcomes (eg, hemoglobin A_1c_ [HbA_1c_], physical function) from baseline to 12 weeks, representing before-and-after health technology–assisted lifestyle self-monitoring. Qualitative data were collected through semistructured participant interviews to help explain the quantitative findings, particularly patterns of engagement in self-monitoring and observed changes in health outcomes, as well as to explore participants’ experiences using health technologies for lifestyle self-monitoring and identify facilitators and barriers to lifestyle modification for diabetes management.

The inclusion criteria were (1) age 60 years and over, (2) self-reported diagnosis of type 2 diabetes with the confirmation of a health care provider’s report or laboratory report following the American Diabetes Association criteria [27], (3) being overweight or obese (BMI ≥27 kg/m²), (4) having a smartphone, and (5) willingness to participate in the study and provide informed consent.

The sample size calculation was based on prior evidence suggesting that a minimum of 12 participants is a rule of thumb for a pilot study [28]. Assuming a 20% dropout rate based on prior studies in older adults [29], this pilot study proposed enrolling 15 older adults with type 2 diabetes into the study.

### Recruitment

Participants were recruited between November 2020 and June 2021 using multiple approaches, including social media platforms (eg, Facebook, Nextdoor), flyers posted at public places (eg, the University of Texas Health Science Center at San Antonio [UTHSCSA]), word of mouth, and mass invites via the UTHSCSA electronic medical record messaging system.

### Ethical Considerations

This study was reviewed and approved by the institutional review board (IRB) at UTHSCSA (IRB application number HSC20200531H) with the statement “Your request to conduct this minimal risk research was approved by Expedited Review on August 11, 2020.” Written informed consent was obtained from each participant prior to study enrollment.

Participant privacy and confidentiality were protected by assigning unique study identifiers and storing data on secure, password-protected institutional servers or in a locked drawer in the principal investigator’s office. Wearable and survey data were deidentified prior to analysis. Only authorized study personnel had access to identifiable information.

Participants received US $15 in compensation for completing data collection at baseline and at the 12-week follow-up, respectively, with up to US $30 in compensation for their participation. Participants were allowed to keep the Fitbit device after the study.

### Procedures

Enrolled participants were instructed to track physical activity and food intake using a Fitbit Inspire HR fitness tracker (Fitbit Inc) and its corresponding smartphone app for over 12 weeks. All tracked data were automatically synchronized to UTHSCSA’s connected health platform. Each participant was provided with a Fitbit fitness tracker at no cost to use.

In addition to standard Fitbit instructions, each participant received a simplified step-by-step instruction handbook with a large font size and pictures created by the study team. The handbook included key points for using the device to track physical activity and food intake. The study team helped each participant in setting up all devices and guided them through the lifestyle tracking process step by step. The study team was available via phone during weekdays for any troubleshooting of technological issues or lifestyle logging questions. The study team could see all participants’ tracked lifestyles through the connected health platform. For those who did not log in for 2 consecutive weeks, the study team would call and remind participants to self-monitor. The study was focused on assessing technology-assisted self-monitoring, and no structured lifestyle education intervention was implemented as part of the study.

### COVID-19–Related Protocol Adaptations

Following the institution’s COVID-19 research implementation protocol, all participants completed a telephone screening one day prior to each scheduled study appointment to assess COVID-19–related symptoms or potential exposure within the preceding 15 days. Study appointments would be rescheduled if participants reported any symptoms or exposures; however, no participants screened positive during the study period. In-person contact was minimized whenever possible, and mask wearing was encouraged but not required during in-person visits. All equipment used for data collection was sanitized after each participant visit.

### Data Collection

Demographic characteristics (eg, age, race or ethnicity, education) were collected at baseline using a survey questionnaire. Health outcome data were collected at baseline and at 12 weeks at UTHSCSA School of Nursing by trained research staff.

#### Feasibility Measures

Feasibility was assessed using recruitment and retention rates, completion of study procedures, adherence to wearable-based self-monitoring (percentage of days with tracked steps and food logs), and completeness of quantitative outcome data. Adherence to self-monitoring was collected using the Fitbit fitness tracker. Physical activity and dietary self-monitoring data were collected continuously over the 12-week period using the Fitbit device and smartphone app. Fitbit-tracked data were synchronized automatically from the Fitbit device to the Fitbit smartphone app and subsequently transmitted to UTHSCSA’s connected health platform when the participant’s smartphone was connected to the internet. No fixed syncing cadence was imposed; syncing occurred passively and opportunistically based on participant device use and connectivity. The percentage of days with tracked steps (PDWTSs) was calculated as the number of days with step counts greater than “0” divided by the total number of days in the study. Days with zero recorded steps were treated as nonwear or nontracking days and were not included. The percentage of days with food logs (PDWFLs) was calculated as the number of days on which any amount of food intake was logged (any value over “0” was counted as a “yes” for that day) divided by the total number of study days. Outlier trimming was not applied due to the exploratory nature of this pilot study.

#### Health Outcome Measures

Body weight was measured using a calibrated digital scale with participants wearing light clothing. Height was measured using a stadiometer, with participants standing upright and barefoot or wearing light socks. All equipment was sanitized after each use. BMI was calculated as weight in kilograms divided by height in meters squared (kg/m²) [30]. Glycemic control was assessed by HbA_1c_ (%) using fasting blood samples. Physical function was assessed by the Short Physical Performance Battery (SPPB), which assesses gait speed, balance, and chair stand performance [31]. It is important to note that SPPB data were not collected for the first 7 participants at baseline, as the study team aimed to minimize in-person contact during the COVID-19 pandemic.

#### Qualitative Data

At 12 weeks, we also conducted semistructured interviews with each of the 15 participants. An interview guide was developed to assess participants who perceived the challenges and facilitators of using health technologies to self-monitor lifestyle behaviors, as well as challenges and facilitators of focusing on physical activity and healthy eating for diabetes management. A list of questions included in the interview guide is provided in Multimedia Appendix 1. All interviews were audio-recorded and transcribed.

### Data Analysis

Sample characteristics and self-monitored lifestyle data were summarized using frequencies and percentages for categorical variables and mean and SD for continuous variables. Baseline and 12-week health outcomes were compared using paired 2-tailed *t* tests or Wilcoxon signed rank tests for variables that were not normally distributed. Effect sizes were calculated using Cohen *d* to quantify the magnitude of change from baseline, and 95% CIs were calculated for Cohen *d* values. The associations of PDWTSs, PDWFLs, and 7-day mean steps with 12-week HbA_1c_ and 12-week physical function (SPPB) were examined using Pearson correlation analysis or Spearman correlation analysis if not normally distributed. Normality was assessed using the Shapiro-Wilk test. Variables with a *P* value of <.05 were determined to be not normally distributed, including body weight, chair test, and total SPPB score. Given the sample size of the pilot study, missing baseline SPPB-related variables were not imputed. Analyses for balance, gait speed, chair stand, and total SPPB score were conducted using available data from the 8 participants with complete measurements. In small pilot studies, imputation may produce unstable estimates due to limited data for model estimation; therefore, complete-case analysis is commonly recommended when the primary aim is feasibility assessment and preliminary effect estimation [32,33]. Self-monitored data were analyzed using Excel (Microsoft 365). Other quantitative data were analyzed using R (version 4; R Foundation for Statistical Computing); the significance level was set at .05, 2-sided.

Two researchers with training and experience in qualitative methods independently analyzed the qualitative data using a combination of deductive and inductive thematic analysis. First, the data were independently coded using open coding in NVivo 11 (version 11; QSR International) by the study investigator (YD) and a trained research assistant. Discrepancies between coders were discussed, and consensus was reached to ensure consistency. An initial codebook with operational definitions was developed and refined throughout the coding process. Although formal procedures for data saturation were not prospectively defined, all 15 participants enrolled in the study were interviewed. Given the pilot nature of the study and the relatively focused research questions, the interviews yielded recurring themes, suggesting that the data were sufficient to capture key participant experiences. Analytic credibility was supported through investigator triangulation and systematic documentation of coding decisions.

Second, when reviewing the codes, themes were organized using the social ecological model as an analytic framework to examine how facilitators and barriers to technology use and lifestyle modifications are influenced by factors across multiple levels [34]. The social ecological model consists of five levels of factors: (1) societal or policy (factors concerning large-scale issues, such as social and cultural norms, and guiding rules, such as policies or laws), (2) community (factors limited to a specific geographic area, such as neighborhoods), (3) organizational (factors at institutional settings, such as schools and workplaces), (4) interpersonal (factors involved with direct person-to-person interaction), and (5) individual (personal characteristics, such as health and beliefs) [35,36]. The Centers for Disease Control and Prevention uses a 4-level social-ecological model by combining the community and organizational levels into a single category [37]. We adopted this 4-level approach in our analysis. Relationships among factors within the social ecological model levels were explored,

Finally, the qualitative findings were explicitly integrated with the quantitative results to support mixed methods triangulation. This integration facilitated a comprehensive interpretation of how factors from multiple levels could influence self-monitoring behaviors, lifestyles, and observed clinical outcomes.

## Results

### Sample Characteristics

The average age of the study sample was 70.5 (SD 4.8) years (Table 1). Forty percent (6/15) of the sample was female, and 73.3% (11/15) of the study participants self-identified as Hispanic. All participants reported some college education or higher. Over half (10/15, 66.7%) of the participants were obese, and the mean baseline HbA_1c_ level was 7.9% (SD 1.9%) at baseline.

**Table 1.** Baseline characteristics of the 15 study participants.

Variables	Values
Age (y), mean (SD)	70.5 (4.8)
Female, n (%)	6 (40)
Race or ethnicity, n (%)	
Hispanic (yes)	11 (73.3)
Non-Hispanic White (yes)	4 (26.7)
Some college and over (yes), n (%)	15 (100)
Family income (≥US $60,000), n (%)	7 (46.7)
Obesity (yes), n (%)	10 (66.7)
HbA_1c_^a^ (%), mean (SD)	7.9 (1.9)

a HbA_1c_: hemoglobin A_1c_.

### Feasibility

The targeted sample size was successfully enrolled over approximately 6 months (from November 2020 to June 2021) after screening 20 subjects who expressed interest in participation. An additional 6 individuals expressed interest, but they were not screened as the targeted enrollment had been reached. All (n=15, 100%) participants completed both baseline and 12-week follow-up data collection.

All 15 enrolled participants used a Fitbit fitness tracker and its associated mobile phone app to self-monitor their physical activity and dietary intake, although adherence varied. MultimediaAppendices 2^4^ show the Fitbit fitness-tracked data. Over the 12-week study period, most participants tracked their steps daily, with 11 out of 15 participants tracking their steps for more than 90% of the study days. Seven out of 15 participants had an average of 5000 daily steps. Nine out of 15 participants tracked over 60% of days with food logs, and only 4 out of 15 participants tracked their food for over 90% of days. Additional feasibility-related findings are reported in the qualitative findings section.

### Health Outcomes

**Figure 1.**
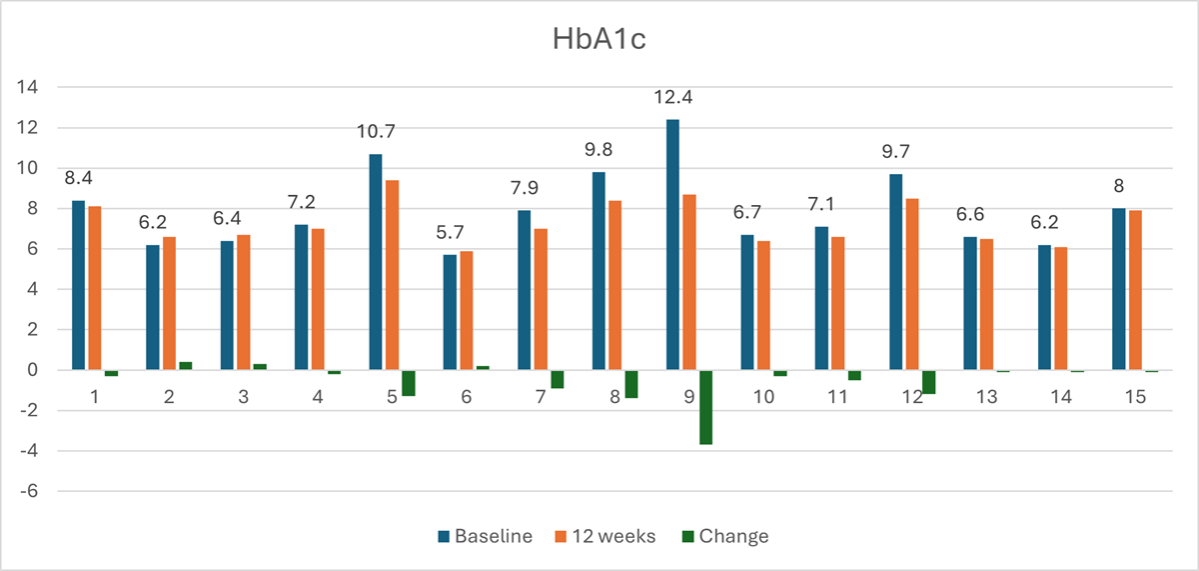
Hemoglobin A_1c_ (HbA_1c_) levels at baseline and 12 weeks for participants.

Figure 1 illustrates HbA_1c_ values at baseline, 12 weeks, and individual-level changes over time for each participant. All participants with a baseline HbA_1c_ greater than 7 had a decrease in HbA_1c_ at the 12-week follow-up (HbA_1c_ changes ranging from −0.1 to −3.7). Those with a baseline HbA_1c_ above 9 showed the greatest reductions (HbA_1c_ changes ranging from −1.2 to −3.7). Participants with baseline HbA_1c_ levels below 7 maintained levels below 7 at the 12-week follow-up.

Table 2 displays the changes in weight, BMI, HbA_1c_, and physical function from baseline to 12 weeks. The mean HbA_1c_ decreased from 7.93 (SD 1.93) to 7.32 (SD 1.08) at 12 weeks, with a statistically significant reduction (mean change −0.61, 95% CI −1.18 to −0.05; effect size −0.49; *P*=.04). Although weight and BMI also showed improvements, these changes were not statistically significant. However, the effect sizes suggest potentially clinically relevant trends. Specifically, body weight had a large effect size of −0.83; BMI also decreased, with a moderate effect size of −0.61. No significant SPPB improvements were found over the study period.

**Table 2.** Health outcomes at baseline and 12 weeks.

Characteristic^a^	Baseline, mean (SD)	12 weeks, mean (SD)	Change^b^ (95% CI)	Effect sizes^c^ (95% CI)	*P* value^d^
Weight (lb)	207.16 (45.07)	205.94 (42.94)	−1.23 (−4.41 to 1.96)	−0.83 (−0.80 to 0.36)	.42
BMI (kg/m^2^)	33.42 (5.42)	33.24 (4.62)	−0.19 (−0.86 to 0.48)	−0.61(−0.74 to 0.42)	.56
HbA_1c_^e^ (%)	7.93 (1.93)	7.32 (1.08)	−0.61 (−1.18 to −0.05)	−0.49 (−1.15 to −0.04)	.04
SPPB^f^					
Balance	3.75 (0.71)	3.71 (0.47)	0.13 (−0.46 to 0.67)	0.15 (−0.69 to 0.99)	.69
Gait	3.75 (0.71)	3.64 (0.49)	−0.13 (−0.66 to 0.41)	0.01 (−1.03 to 0.64)	.60
Chair	2.08 (1.49)	2.37 (1.57)	0.29 (−0.88 to 1.46)	0.15 (−1.05 to 0.62)	.56
SPPB (total)	9.35 (1.99)	9.75 (2.06)	0.40 (−1.03 to 1.83)	0.20 (−0.96 to 0.71)	.74

a Due to COVID-19, balance, gait, chair, and total SPPB scores were not collected at baseline for the first 7 participants; therefore, only 8 participants’ SPPB data are presented in the table.

b Changes represent mean differences (95% CI) from baseline to 12 weeks.

c Effect sizes are reported as Cohen *d* (95% CI).

d *P* values were calculated using paired 2-tailed *t* tests or Wilcoxon signed rank tests, as appropriate.

e HbA_1c_: hemoglobin A_1c_.

f SPPB: Short Physical Performance Battery (higher scores indicate better performance).

Table 3 demonstrates the correlations of Fitbit-tracked data with average daily steps and health outcomes (BMI, HbA_1c_, and SPPB) at 12 weeks. PDWTSs, PDWFLs, and average daily steps showed expected trends in their associations with BMI, HbA_1c_, and SPPB, but not all of the correlations were statistically significant. PDWTSs and PDWFLs showed a high correlation with each other (*r*=0.71; *P*=.002). PDWFL was significantly correlated with lower HbA_1c_ levels (*r*=−0.53; *P*=.04). In addition, a negative relationship between BMI and SPPB was noted (*r*=−0.57; *P*=.03), indicating that higher BMI was related to lower physical function.

**Table 3.** Correlations of Fitbit-tracked data with health indicators at 12 weeks^a^.

	PDWTS^b^	PDWFL^c^	Steps	BMI	HbA_1c_^d^	SPPB^e^^,^^f^
PDWTS	1	0.71^g^	0.27	−0.10	−0.38	0.24
PDWFL	0.71^g^	1	0.43	−0.08	−0.53^h^	0.32
Steps	0.27	0.43	1	0.07	−0.30	0.26
BMI	−0.10	−0.08	0.07	1	−0.06	−0.57^h^
HbA_1c_	−0.38	−0.53^g^	−0.30	−0.06	1	0.01
SPPB	0.24	0.32	0.26	−0.57^g^	0.01	1

a All 15 participants’ 12-week SPPB data were included in the analysis.

b PDWTS: percentage of days with tracked steps.

c PDWFL: percent of days with food logs.

d HbA_1c_: hemoglobin A_1c_.

e SPPB: Short Physical Performance Battery (higher scores indicate better performance).

f Spearman correlation was used for SPPB due to nonnormal distribution; Pearson correlation was used for all other variables.

g *P*<.01 level.

h *P*<.05 level.

### Qualitative Findings

**Figure 2.**
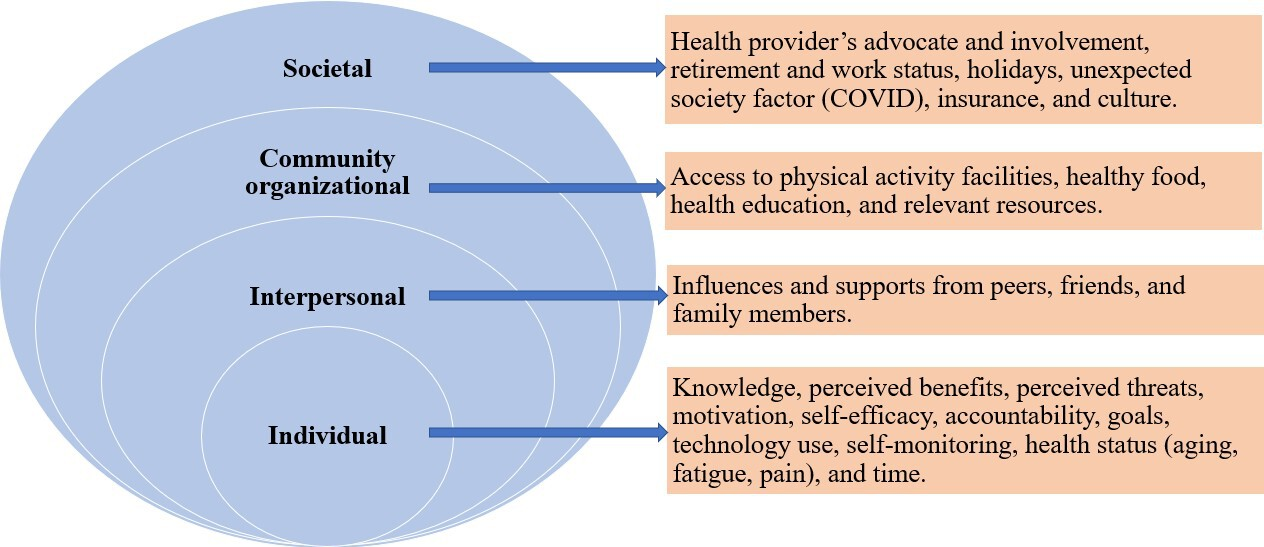
A conceptual framework of factors to promote healthy lifestyles for diabetes management in older adults with type 2 diabetes: an integration of the social ecological model with themes and subthemes of interview data.

The qualitative findings demonstrated participants’ experiences with technology-assisted lifestyle monitoring and provided insights into factors influencing the feasibility, engagement, and sustainability of diabetes self-management behaviors. Barriers and facilitators to lifestyle behaviors for diabetes management were identified across multiple ecological levels, including individual, interpersonal, organizational/community, and societal factors (Figure 2).

The factors at different levels influence both the feasibility of technology use and healthy lifestyle behaviors. For instance, an individual’s self-monitoring practices and motivation may be strengthened by interpersonal support from family and friends, while access to community programs and health education further reinforces these behaviors. Additionally, societal-level influences, such as health care policies and cultural norms, can either enable or hinder diabetes management by shaping access to resources and behavioral expectations. We described the factors at each level below. The participant identification number and gender are provided following each example quote.

Several factors were found at the individual level, such as knowledge, perceived benefits, goals, self-monitoring, and health status. Participants mentioned that their self-discipline, motivation, self-efficacy, and goal setting influenced their ability to engage in physical activity and maintain a healthy diet. However, challenges such as fatigue, pain, and time constraints hindered adherence to recommended behaviors. Examples of participant quotes are provided below.

*So, for two years now I have been doing 10,000 steps every day. Yeah, so the way I do it to get it done is that after breakfast, I do 3,000 steps for sure. Then after lunch, I do the next 3000 and after dinner I have to get them done. So, I usually have a lot less than what I need after dinner because I really do more than 3000 each time. But I have it in my head that I have to have. I divide it because you can’t do it all at the end of the day, you won’t get it done. So, you have to be disciplined to do it … (what motivated me is that), the XX (a health condition) that I have, and the doctor told me that I was going to be on lifetime pain medications, and I didn’t want to do that. And he said that walking is good.*

[P3, female]

*That’s the pain I’m talking about. I have trouble, like I couldn’t get out of that chair because of the pain in my knees and so it’s hard for me to walk upstairs because of the pain in the knees. It’s hard to walk, it’s hard to get off the chair, so then, so then I don’t want to get out of the chair because it just hurts too much, you know …*

[P5, male]

*I think it (Fitbit fitness tracker) keeps it (blood glucose) from getting higher. You know I keep track of everything else, so when I overeat or under exercising, and it helps to keep my sugar more or less. So far, I have been doing good to keep it (HbA_1c_) under seven.*

[P 2, male]

In addition, at the individual level, older adults frequently report challenges with using Fitbit and its paired smartphone app to track food intake, primarily due to limited technological literacy. Some participants found it difficult to navigate the app or use it consistently in real time. As a result, many resorted to writing down their meals on paper or a calendar first and then manually transferring the information to the app later.

*… So that maybe somebody like her age or my daughter’s age or a 40-year-old person because they’re so used to technology that might be OK, but for a 70-year-old woman it wasn’t OK. So, I don’t have a lot of information on this thing … I like having it because when I look I can I have it on my phone, I can see my steps …*

[P6, female]

*That (logging food) was the hardest part because a lot of times what I would do is at first, I had trouble keeping track of it, but then I would just start writing it down because I would forget and if I didn’t do it that day and by the next day, I would forget … Yes, when I ate and what I ate and so forth. So, usually I would put it on, like on a calendar and then I would go back on the Fitbit and enter all the data in there.*

[P2, male]

At the interpersonal level, diabetes management behaviors could be influenced by others, such as family members and friends. For instance, challenges involved managing family dynamics around dietary preferences. While family and friends provided emotional support and encouragement, conflicting food choices and social eating norms sometimes made it difficult to sustain healthy eating habits. Peer support and accountability played a role in promoting physical activity, with some participants relying on social networks to stay engaged.

*… She (daughter) and my husband don’t have to worry about what they consume, let me put it that way, and I do. Which means we are having two separate dinners and sometimes I don’t want to make two separate dinners. So, and he’ll make dinner, but he forgets about the fact that I have to watch what goes into that, and it’s harder, dinner is harder.*

[P1, female]

*And I really have noticed that, when I first started, I was first doing my steps, but was crying all throughout the time, because my pain level would shoot up. And I have this friend, who told me, look I will be on the phone with you while you are walking, that way you are distracted. So, she would stay on the phone with me, all the time that I was crying in pain, and she would encourage me, you are doing okay, go ahead, we will just change the subject.*

[P3, female]

*I have to keep up with my great grandson because he’s the one showing me stuff on the phone and on the computer.*

[P2, male]

At the community and organizational levels, participants mentioned access to physical activity programs and healthy food options as important facilitators. However, logistical barriers, such as the limited availability of facilities, transportation challenges, and financial constraints, were reported. Access to health education and relevant community resources was also noted as a crucial factor influencing lifestyle behaviors.

*I live on the other side of town and dealing with the traffic is not very good, especially at certain times.*

[P2, male]

*I’m taking a balance class, they just restarted it because of the COVID, they could not allow instructors to come into the building, but they started back up in April. So, I just started, and I have attended two classes, every Monday at 10:00.*

[P3, female]

At the societal level, the role of health providers as advocates and sources of guidance was emphasized. Participants expressed that provider involvement and encouragement significantly impacted their motivation to adhere to diabetes management behaviors. Broader societal factors, such as holidays, cultural norms, insurance policies, and unexpected societal disruptions such as the COVID-19 pandemic, also shaped lifestyle behaviors by affecting access to care, dietary patterns, and social routines.

*…There again he (doctor) just talks about the medication, he doesn't talk about that the medical or what do you call it? Diabetes education or something like that.*

[P5, male]

*And so they say well we have this kind of cake and this kind of cake and said well bring them both for her so she’s 70, so I’m like OK. Well I can’t eat this kind of stuff but I took a piece of each, you know. Then, my son taking out that day and I mean I was celebrating my birthday for more than a month so there was a lot of extra goodies.*

[P11, female]

*Well yeah like when I was, we get together for Mother’s Day for the whole group for my wife’s children or like my children and my in-laws and you know, we went out to eat, you know, Mexican food. And you have this and have a drink and you know, I got home and like blood sugar levels were through the roof, you know?*

[P5, male]

*And the so-called breakthrough cases too. In fact, my husband and I both had RSV and so we thought we had COVID at first and with that and it was mostly not go anywhere but yeah not being able to go to water aerobics. It really affected us because the pools aren’t even open, like at the gyms and stuff. And I don’t exercise, it definitely affects my blood sugar.*

[P12, female]

### Integrated Quantitative and Qualitative Findings

Participants who demonstrated consistent step tracking and dietary logging described strong motivation, clear goal-setting strategies, and reinforcement from interpersonal support, which aligned with observed associations between food logging frequency and lower HbA_1c_ levels (eg, participant 3). Conversely, qualitative barriers related to limited digital literacy, pain, fatigue, and competing social demands provided explanatory context for inconsistent dietary logging and the modest glycemic outcomes. Multilevel influences, such as interpersonal encouragement and access to community resources, emerged as key factors that supported engagement with self-monitoring despite technological challenges, highlighting conditions under which technology-assisted lifestyle self-monitoring may be more feasible and acceptable among older adults with type 2 diabetes.

## Discussion

### Principal Findings

This mixed methods study explored the feasibility and potential health benefits of using wearable and mobile devices for older adults to manage type 2 diabetes and identified potential strategies to support lifestyle modification for diabetes management. The study proved to be feasible even during the challenging period when COVID-19 was still actively circulating in the community. The quantitative results highlight the potential benefits of digital devices in supporting self-monitoring and improving glycemic control among older adults with type 2 diabetes. A significant reduction in HbA_1c_, as well as the positive association between HbA_1c_ and the percentage of days with food logs, suggests that consistent tracking of food intake plays a critical role in managing blood glucose levels. These findings align with previous research emphasizing the importance of health technologies and lifestyle modifications in diabetes management. The qualitative findings underscore the multilevel challenges and facilitators of achieving sustained behavioral changes. The findings suggest that multilevel interventions, which address individual motivation and self-efficacy, family and peer support, community resources, and health care involvement, are necessary for the comprehensive management of diabetes.

### Feasibility and Self-Monitoring

This pilot study demonstrated the feasibility of using technology-assisted lifestyle monitoring for older adults with type 2 diabetes. The strong enrollment rate and 100% (n=15) retention at 12 weeks indicate high engagement and acceptability, key indicators of feasibility in pilot research [38]. All participants used a Fitbit fitness tracker and its companion app for 12 weeks to monitor physical activity and diet with varied adherence. Prior studies have shown that older adults are capable of using wearable devices for physical activity tracking when provided with proper training and support [39,40]. However, dietary logging was found to be less consistent, with only 2 participants having logged food intake on over 90% (81/90) of days. This is consistent with the literature showing that food tracking can be cognitively demanding and burdensome, particularly for older adults [41]. This suggests the need to explore approaches to ease the food logging tasks and provide tailored support strategies. Additionally, strategies that offer ongoing, convenient, and hands-on support may help mitigate the challenges of low digital literacy among older adults.

### Health Outcomes

The observed reduction in HbA_1c_ levels in this pilot study should be interpreted cautiously, given the small sample size and short duration. However, the findings are consistent with prior research suggesting that self-monitoring may support diabetes self-management [42]. Although we did not provide any additional interventions beyond instructing participants on how to use the Fitbit fitness tracker and its paired smartphone to track physical activity and food intake, our findings are consistent with previous studies suggesting that self-monitoring is associated with improved physical activity and dietary adherence, leading to improved glycemic outcomes in individuals with type 2 diabetes [43,44]. Although the average daily step counts were not significantly associated with BMI, glycemic control, or physical function, trends were in the expected directions. Additionally, emerging evidence also indicates that the timing of physical activity may influence glucose regulation, with postmeal or evening activity potentially offering advantages in glycemic management [43-45]. These findings highlight areas for further investigation, particularly in larger and more rigorously designed studies.

### Qualitative Findings

The qualitative findings helped contextualize variability in self-monitoring adherence, lifestyles, and health outcome trends observed in the quantitative data. Qualitative data reinforce the view that self-monitoring and lifestyle behaviors are shaped by a complex interplay of individual, interpersonal, organizational or community, and societal factors, echoing the social ecological model [34]. At the individual level, factors such as motivation, goal setting, and self-monitoring emerged as crucial determinants, yet physical limitations, pain, and fatigue were notable barriers, similar to what has been reported among older adults with chronic disease [45]. Social and familial contexts further influenced dietary behaviors and physical activity, with both supportive and conflicting dynamics observed. Previous studies have also shown that family engagement can enhance or impede diabetes self-care depending on the alignment of goals and routines [46]. Interventions for diabetes management, involving support from family members, peers, or friends, could be promising.

At the community level, access to programs and transportation challenges mirrored structural barriers commonly faced by older adults. Organizational support, such as restarting community exercise classes, was valued and perceived as helpful. These insights support broader calls for age-friendly and community-based programs, such as exercise classes and diabetes education for older adults [47]. On the societal level, participants highlighted the role of health care providers as motivators, though some noted insufficient attention to lifestyle counseling, suggesting an opportunity to better integrate behavioral support into health care settings [48] or to bridge health care and community services [49,50]. External societal events, such as the COVID-19 pandemic, were cited as disruptive to routine care and physical activity access, underscoring the need for adaptable and resilient health promotion strategies in the face of such events.

### Limitations

This study has several limitations that should be acknowledged. The study had a small sample size (n=15) using a 1-arm design, so the statistical power to detect significant changes was limited. In addition, SPPB data were not collected at baseline for the first 7 participants due to COVID-19 restrictions, which further constrained the interpretation of physical function outcomes. As a result, several observed trends may not have reached statistical significance. The lack of a control group limits the ability to attribute observed changes directly to the intervention. Future studies should include randomized control groups to strengthen causal inferences. The 12-week intervention period may have been insufficient to observe meaningful changes in physical function and other long-term health outcomes. Longer follow-up periods are needed to assess the sustainability of the observed improvements. The study population consisted predominantly of Hispanic participants, which may limit the generalizability of the findings to other ethnic groups. Additionally, participants were required to own smartphones, which might have introduced selection bias toward individuals who were more technologically engaged and health motivated. The study participants were of relatively moderate-to-high socioeconomic status, which may further limit generalizability. While wearable devices provide objective data, their health benefits may also depend on participants’ compliance and engagement with self-monitoring activities. Variability in participants’ adherence may have influenced the outcomes. The study defined PDWFLs and PDWTSs using the “any nonzero value” as a valid entry, which may not accurately capture the true level of engagement in physical activity and dietary intake. Finally, although all study participants were interviewed, the extent to which true data saturation was achieved may be limited, which may in turn constrain the robustness of the qualitative findings.

### Conclusions

Using technology-assisted self-monitoring of lifestyle behaviors was feasible among a select group of older adults with type 2 diabetes who were smartphone users and relatively well educated. Although favorable within-subject signals were observed for glycemic control and related outcomes, these findings should be interpreted cautiously, given the study’s single-arm design and potential selection bias. Importantly, the results highlight that the benefits of technology-assisted self-monitoring are likely contingent on broader contextual factors, including digital and health literacy, access to devices, and the availability of interpersonal, organizational, and social support. To inform equitable and scalable lifestyle–based diabetes management interventions, future studies should investigate more diverse study populations and include older adults with lower digital literacy or limited access to technology. Additionally, qualitative findings revealed that multilevel lifestyle interventions facilitated by health technologies to enhance lifestyle-based diabetes self-management hold promise. However, the use of technology presents both opportunities and challenges. Further research is warranted to examine how health technologies can be integrated with multilevel strategies to support long-term, sustainable lifestyle-based diabetes management among older adults with type 2 diabetes.

## Supplementary material

10.2196/79591 Multimedia Appendix 1 Semistructured interview guide.

10.2196/79591 Multimedia Appendix 2 Fitbit-tracked data in percent of days with tracked steps.

10.2196/79591 Multimedia Appendix 3 Fitbit-tracked data in percent of days with food logs.

10.2196/79591 Multimedia Appendix 4 Fitbit-tracked data in average daily steps.

## Abbreviations

HbA_1c_: hemoglobin A_1c_
PDWFL: percentage of days with food log
PDWTS: percentage of days with tracked step
SPPB: Short Physical Performance Battery
UTHSCSA: University of Texas Health Science Center at San Antonio
