# Supplementary material for: Feasibility of Technology-Assisted Lifestyle Self-Monitoring in Older Adults With Type 2 Diabetes: Mixed Methods Pilot Study
Source: JMIR Form Res. 2026 Jun 3;10:e79591. doi: 10.2196/79591 (PMC13232604; doi:10.2196/79591)
Supplement: Multimedia Appendix 1 [file formative-v10-e79591-s001.docx]

**Appendix I. Semi-Structured Interview Guide**

**Introduction**

1. Welcome and thank you for participating.
2. Brief overview of the study

- Review the objective of the study: to understand how to improve diabetes care, explore technology use for diabetes self-management, and identify challenges and facilitators related to healthy lifestyle behaviors and complication prevention among older adults living with diabetes.
- There are no right or wrong answers; we are interested in different perspectives and experiences.
- The session will be audio recorded.
- Every effort will be made to maintain confidentiality. Responses will be de-identified after recording, and participants are also encouraged to maintain confidentiality by not sharing discussions outside the session.

**Section 1. Experiences Living with Diabetes and Diabetes Management**

1. Was there a particular time when managing your diabetes felt especially challenging?
2. Did your doctor talk with you about preventing diabetes complications (e.g., kidney disease)?
3. What are your overall health concerns related to diabetes?

**Section 2. Health Technology Use and Lifestyle Self-Monitoring**

1. Have you ever used wearable devices or other health technologies to track your lifestyle behaviors or support diabetes management?
2. How comfortable are you using these devices?
3. What challenges or problems have you encountered when using these devices?
4. During the past 3 months, were you able to wear or use the device that we gave to you consistently? Why or why not?
5. Do you have any concerns or questions about logging food or tracking diet? What are the concerns?
6. Do you have any concerns about using wearable or digital health devices? What are the concerns?
7. Do you think tracking your lifestyle behaviors is helpful? Why or why not?
8. Has the COVID-19 pandemic affected your physical activity or diet? If yes, how and why?

**Section 3. Physical Activity and Healthy Eating**

1. What are the main challenges to engaging in physical activity?
2. What types of physical activity do you currently do?
3. What helps or motivates you to stay physically active?
4. What are the main challenges to maintaining a healthy diet?
5. What helps or motivates you to eat healthy food?

**Section 4. Protocol and Data Collection Feasibility**

1. Is the survey too long or difficult to understand?
2. Did you experience any difficulties with the urine or blood sample collection procedures?

**Closing**

1. Do you have any additional comments or questions for us?
2. Thank you again for your participation.
