# Supplementary figures and images for: Feasibility of Technology-Assisted Lifestyle Self-Monitoring in Older Adults With Type 2 Diabetes: Mixed Methods Pilot Study
Source: JMIR Form Res. 2026 Jun 3;10:e79591. doi: 10.2196/79591 (PMC13232604; doi:10.2196/79591)

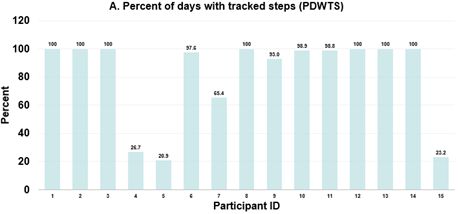

Supplement: Multimedia Appendix 2 [file formative-v10-e79591-s002.png]

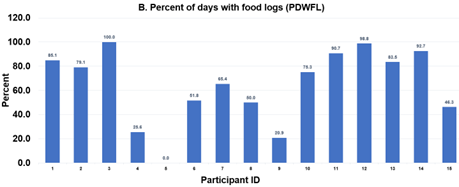

Supplement: Multimedia Appendix 3 [file formative-v10-e79591-s003.png]

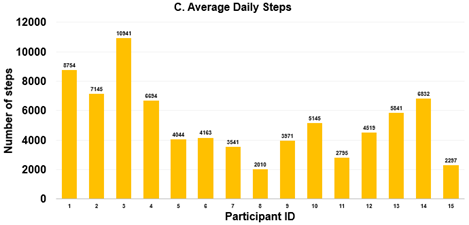

Supplement: Multimedia Appendix 4 [file formative-v10-e79591-s004.png]
